# Supplementary material for: Assay System for Simultaneous Detection of SARS-CoV-2 and Other Respiratory Viruses
Source: Diagnostics (Basel). 2021 Jun 13;11(6):1084. doi: 10.3390/diagnostics11061084 (PMC8231941; doi:10.3390/diagnostics11061084)
Supplement: Supplementary file 1 [file diagnostics-11-01084-s001.zip › diagnostics-1238073-supplementary.pdf]

Table S1. Eight strains of different lineages of SARS-CoV-2 used to evaluate the analytical specificity

Company	Cat No.	Lineage	Description	2019-nCoV	STANDARD M	SC2FabR	Comment
NCCP	43381	B.1.1.7	United Kingdom	SARS-CoV-2	SARS-CoV-2	SARS-CoV-2	Match
	43382	B.1.351	South Africa	SARS-CoV-2	SARS-CoV-2	SARS-CoV-2	Match
	43383	P.2	Brazil	SARS-CoV-2	SARS-CoV-2	SARS-CoV-2	Match
	43384	B.1.427	USA (CA)	SARS-CoV-2	SARS-CoV-2	SARS-CoV-2	Match
	43385	B.1.429	USA (CA)	SARS-CoV-2	SARS-CoV-2	SARS-CoV-2	Match
	43386	B.1.525	International	SARS-CoV-2	SARS-CoV-2	SARS-CoV-2	Match
	43387	B.1.526	USA (NY)	SARS-CoV-2	SARS-CoV-2	SARS-CoV-2	Match
Twist Bio	79683	P.1	Brazil	SARS-CoV-2	SARS-CoV-2	SARS-CoV-2	Match

Strain description, cited by the PANGO lineages from which the SARS-CoV-2 strain was acquired, is shown. Abbreviations: SARS-CoV-2, severe acute respiratory syndrome-related coronavirus 2; NCCP, National Culture Collection for pathogens.

Table S2. Eighteen samples used to evaluate the clinical performance of the kit for simultaneous detection of co-infection

Sample	2019-nCoV	RP1	SC2FabR	Comment
A	SARS-CoV-2	Flu A-H1pdm09	SARS-CoV-2, Flu A	Match
B	SARS-CoV-2	Flu A-H1pdm09	SARS-CoV-2, Flu A	Match
C	SARS-CoV-2	RSV A	SARS-CoV-2, RSV	Match
D	SARS-CoV-2	RSV A	SARS-CoV-2, RSV	Match
E	SARS-CoV-2	-	SARS-CoV-2	Match
F	SARS-CoV-2	-	SARS-CoV-2	Match
G	SARS-CoV-2	-	SARS-CoV-2	Match
H	SARS-CoV-2	-	SARS-CoV-2	Match
I	SARS-CoV-2	-	SARS-CoV-2	Match
J	SARS-CoV-2	-	SARS-CoV-2	Match
K	SARS-CoV-2	-	SARS-CoV-2	Match
L	SARS-CoV-2	-	SARS-CoV-2	Match
M	SARS-CoV-2	-	SARS-CoV-2	Match
N	SARS-CoV-2	-	SARS-CoV-2	Match
O	SARS-CoV-2	-	SARS-CoV-2	Match
P	SARS-CoV-2	-	SARS-CoV-2	Match
Q	SARS-CoV-2	-	SARS-CoV-2	Match
R	SARS-CoV-2	-	SARS-CoV-2	Match

Abbreviations: SARS-CoV-2, severe acute respiratory syndrome-related coronavirus 2; Flu A-H1pdm09, influenza A virus subtype H1pdm09; RSV, respiratory syncytial virus.
